# Non-absorbable apple procyanidins prevent obesity associated with gut microbial and metabolomic changes

**DOI:** 10.1038/srep31208

**Published:** 2016-08-10

**Authors:** Saeko Masumoto, Akari Terao, Yuji Yamamoto, Takao Mukai, Tomisato Miura, Toshihiko Shoji

**Affiliations:** 1 National Institute of Fruit Tree Science, National Agriculture & Food Research, Tsukuba, Ibaraki, Japan; 2 Department of Animal Science, School of Veterinary Medicine, Kitasato University, Towada, Aomori, Japan; 3 Hirosaki University Graduate School of Health Sciences, Hirosaki, Aomori, Japan

## Abstract

Several studies have suggested that flavan-3-ols/procyanidins are associated with a reduced risk of developing obesity and metabolic syndrome. However, the role of highly polymeric procyanidins (PP), which are major non-absorbable flavonoids, in the biological effects, is not completely understood. Here, we show that 0.5% PP administration for 20 weeks alleviated obesity and regulate expression of genes related to lipid metabolism in C57BL/6J mice fed a high-fat/high-sucrose diet. PP-treatment attenuated weight gain and inflammatory effects including lipopolysaccharide and gut permeability. Additionally, metabolic urine profiling using high-performance liquid chromatography–quadrupole time-of-flight/mass spectrometry demonstrated that PP-treatment decreased the levels of endogenous metabolites associated with insulin resistance. Furthermore, microbial 16S rRNA gene sequencing of the cecum demonstrated that PP administration markedly decreased the *Firmicutes*/*Bacteroidetes* ratio and increased eight times the proportion of *Akkermansia*. These data suggest that PPs influence the gut microbiota and the intestinal metabolome to produce beneficial effects on metabolic homeostasis.

Several epidemiological studies have suggested that diets rich in fruit are associated with a reduced risk of developing chronic disease^1^,^2^,^3^. Fruits, including apples, provide major sources of polyphenols, dietary fiber, and carotenoids, in addition to other nutrients. Studies using animals and human subjects have demonstrated that fruit polyphenols prevent obesity, type 2 diabetes (T2D), cardiovascular disease, and cancer^4^,^5^. Fruit polyphenols likely promote beneficial effects by scavenging free radicals, regulating gene expression, and altering signal transduction in target cells and tissues^6^,^7^.

Polyphenols are ubiquitous secondary metabolites in fruits and plants. Fruit polyphenols can be divided into different structural subclasses, including phenolic acids and flavonoids. Flavan-3-ols/procyanidins are the major flavonoids in apple, grape, and cranberries. Flavan-3-ols are a subclass of flavonoids that includes the simple monomers (+)-catechin and its isomer (−)-epicatechin. Procyanidins, commonly known as condensed tannins, are oligomers and polymers of flavan-3-ol through the interflavanoid linkage of 4 → 8 or 4 → 6 (B-type) (Fig. 1a)^8^. Flavan-3-ols/procyanidins are characterized by their degree of polymerization and their molecular masses, which range from <100 Da for phenolic ompounds to >30,000 Da for highly polymeric compounds^8^.

Ingestion of procyanidins significantly reduce weight gain, decrease adipose tissue mass, and ameliorate insulin tolerance in various animal models and human studies^9^. Indeed, some studies suggest that the consumption of procyanidin-rich foods affects the expression of key genes involved in the regulation of anti-oxidative enzymes and of glucose and lipid metabolism^10^,^11^. Furthermore, determination of flavan-3-ol/procyanidin bioavailability is a prerequisite to understanding their effects on human health. Knowledge of the bioavailability of low-molecular weight flavan-3-ols and oligomeric procyanidins (OPs) has significantly progressed in the last decade^12^. Animal and human studies reported lower absorption of flavan-3-ols/procyanidins in comparison with other flavonoids and procyanidin tetramers observed in the plasma; most highly polymeric procyanidins (PPs) larger than pentamers are not absorbed^13^,^14^. Mechanisms by which non-absorbable PPs inhibit obesity and correct energy homeostasis are poorly understood.

Recent studies demonstrate that the gut microbiota represents a major environmental factor that contributes to obesity, diabetes, and metabolic syndrome. Decreased obesity in germ-free mice demonstrated that the gut microbiota affects host energy regulation and nutrient acquisition and improves insulin sensitivity and glucose tolerance^15^,^16^. The relative abundance of *Firmicutes* in genetically obese C57BL/6J (*ob*/*ob*) mice increased in comparison with that of normal lean (*ob*/+ and +/+) mice, whereas the relative abundance of *Bacteroidetes* decreased^17^. Studies in obese human fed a fat- or carbohydrate-restricted low calorie diet revealed that these subjects have a lower abundance of *Bacteroidetes* and a higher abundance of *Firmicutes* in their gut microbiota; the abundance of *Bacteroidetes* progressively increased over the course of the study^18^. In addition, there is growing evidence indicating that endotoxic lipopolysaccharide (LPS), derived from gram-negative bacteria, and chronic low-grade inflammation are associated with obesity and insulin resistance^19^. Endotoxic inflammation is a result of increased intestinal permeability to LPS, caused by disruption of the gut barrier function and by increased activity of LPS transporter^20^. Therefore, diet significantly influences the gut microbiota directly or via interactions with dietary components and represents a new target for treatments aimed at preventing and treating diseases.

Recently, cranberry extracts and grape procyanidin mixtures with other polyphenols have been reported to modulate the gut microbiota and improve obesity and diabetes in animal model^21^,^22^. However, major questions remain in relation to the health benefits of non-absorbable PPs and the microbial and metabolic signatures associated with PP administration in a model of diet-induced obesity. In the present study, we investigated the effects of non-absorbable PPs on host energy homeostasis associated with the gut microbial and metabolic signatures to demonstrate that non-absorbable PPs improve host lipid metabolism and suppress diet-induced obesity in C57BL/6J mice fed high-fat/high-sucrose (HFHS) diet. These results suggest that differences in the chemical structures of procyanidins affect immunological and nutritional homeostasis in the host via the gut microbiota and the resultant metabolites.

## Results

### PP administration improves lipid metabolism in obese mice

To determine whether PP ameliorated obesity and improved lipid metabolism, we administered either absorbable OPs or non-absorbable PPs to HFHS-fed mice. PP administration significantly blunted the body weight gain of the HFHS-fed mice during the 20-week administration period in comparison with that of the untreated and OP-treated HFHS-fed mice (Fig. 2a). Importantly, the food and water intake of the groups did not differ significantly (Fig. 2b,c). PP administration significantly reduced liver weight (Fig. 2d). Interestingly, non-absorbable PPs prevented visceral and subcutaneous adipose fat gain (Fig. 2e,f), suggesting that PP bioavailability was not an important determinant of lipid homeostasis. Additionally, PP administration to HFHS-fed mice alleviated their hyperglycemia (Fig. 2g), hypertriglyceridemia (Fig. 2h), and hypercholesterolemia (Fig. 2i). Moreover, PP administration also decreased the levels of serum inflammatory cytokines (tumor necrosis factor-alpha (TNF-α), and interleukin-6 (IL-6)) (Fig. 3a). Interestingly, the HFHS diet-fed mice showed a four-fold increase in the level of LPS, as compared with ND-fed mice; this effect was attenuated in the PP-treated HFHS-fed mice, where the LPS level improved to that observed in the ND-fed mice (Fig. 3a). In addition, the expression of *Tnfa* level in liver, adipose tissue and ileum were decreased significantly by PP treatment (Fig. 3b). In addition, PP-treatment attenuated intestinal permeability (upregulation of ZO-1 gene (*Tjp1*) and occludin gene (*Ocln*) expression) of the HFHS-fed mice (Fig. 3c). Interestingly, expression of LPS receptor *Tlr4* and *Cd14* in liver decreased in the PP-treated HFHS-fed mice (Fig. 3d).

### Influence of PPs on expression of genes related to lipid metabolism in liver

PP administration significantly improved mRNA expression levels of fatty acid synthase (*Fasn*) and 3-hydroxy-3-methylglutaryl-coenzyme A reductase (*Hmgcr*), as compared to the levels in untreated HFHS-fed mice (Fig. 3e). The PP-treated mice showed increased mRNA expression of lipogenesis markers, including peroxisome proliferator activated receptor alpha (*Ppara*), AMP-activated protein kinase (*Prkaa1*), carnitine palmitoyltansferase 1a (*Cpt1a*) (Fig. 3e) as compared to the levels observed in the untreated HFHS-fed mice.

### Urinary metabolomics

We assessed the urinary metabolome using high performance liquid chromatography-quadruple time of flight mass spectrometry (HPLC-QTOF/MS) to investigate the gut microbiota of the HFHS-fed mice following PP administration. Principal component analysis-discriminant analysis (PCA-DA) score plots of these profiles, obtained in negative ionization mode, clearly revealed diet-procyanidin specific clustering. The first two components (PD2 and PD3) of the PCA-DA model accounted for 66.9% of the individual variation (Fig. 4a). The characteristic metabolites responsible for the discrimination between the study groups were revealed using a loading plot (Supplementary Fig. 1). Moreover, hierarchical clustering analysis of the 2992 altered metabolites (*p* < 0.01 relative to HFHS-fed mice, *t*-test) showed separate clusters among the treatment groups (Fig. 4b). Several spectral peaks detected in the loading plots were assigned using our in-house library and online metabolome databases. The assigned metabolites were classified into several groups based on retention time and detected and calculated theoretical masses: 12 flavan-3-ol conjugates and 62 phenolic acid conjugates degraded by the gut microbiota and 21 endogenous metabolites (Supplementary Table 1). We were able to distinguish metabolites of flavan-3-ol conjugates and phenolic acid conjugates, degraded from flavan-3-ols by the gut microbiota, in the OP-treated HFHS-fed mice from the other groups, consistent with the reported metabolic pathway for flavan-3ols^23^,^24^. Twenty endogenous metabolites (e.g., bile acid derivatives from lipid metabolism and aromatic amino acid derivatives from tryptophan and tyrosine metabolism) were putatively identified in urine of the PP-treated HFHS-fed mice. In particular, the levels of several bacterial products of aromatic amino acid breakdown, including *p*-cresol sulfate, phenol sulfate, and indole derivatives, decreased in the PP-treated HFHS-fed mice (Fig. 4c).

### Effects of PP administration on the gut microbiota in the cecum

To determine whether PP affected the gut microbiota in the HFHS-fed mice, microbial composition was assessed using Illumina-based 16S rRNA sequencing of cecum DNA from the mice in each group. The UniFrac-based principal co-ordinate analysis (PCoA) plot showed that PP administration had a substantial effect on the gut microbial composition in the HFHS-fed mice (Fig. 5a). At the phylum level, the proportion of sequences assigned to *Firmicutes* significantly decreased in the PP-treated HFHS-fed mice, whereas the proportion of reads assigned to *Bacteroidetes* and *Verrucomicrobia* slightly increased (Fig. 5b, Supplementary Table 2). The *Firmicutes*/*Bacteroidetes* ratio of the PP-treated HFHS-fed mice improved to the level observed in the ND-fed mice (Fig. 5c). Moreover, hierarchical clustering analysis of the 35 altered operational taxonomic units (OTUs) (as percentage of the total microbiota) showed separate clusters in the treatment groups at the genus level (Fig. 5d). A number of notable changes were observed at the genus level (Supplementary Table 3). Increased proportions of sequences assigned to the *Adlerceitzia*, *Roseburia*, *S24-7, Bacteroids, Anaerovorax, rc4-4*, and *Akkermansia* genera were observed in the PP-treated HFHS-fed mice over that of the untreated HFHS-fed mice. Interestingly, PP-administration was associated with an increase in the proportion of reads assigned to *Akkermansia*, a predominant genus in *Verrucomicrobia* (Fig. 5e). In contrast, the proportion of reads assigned to *Clostridium*, *Lachnospiraceae*, and *Bifidobacterium* was reduced by PP administration to HFHS-fed mice.

## Discussion

Obesity and metabolic syndrome are caused by various lifestyle and environmental factors, including a lack of exercise, stress, diet, and genetic factors. Among these factors, fat-enriched diet are involved in the changing response to insulin and increasing chronic inflammation. Apple procyanidins (APCs), the major flavonoids in apples, regulate sugar and lipid homeostasis^25^,^26^. Our results suggest that administration of absorbable OPs affects the expression level of key genes involved in the regulation of oxidative stress and glucose and lipid metabolism, consistent with previous studies^10^,^11^. Metabolomics analyses of OP-treated mice revealed increased levels of exogenous flavan-3-ol conjugates and their related low-molecular weight phenolic acid metabolites in the gut microbiota (Supplementary Table 1)^27^,^28^. Recently, it was reported that some phenolic acid metabolites may have biological antioxidant^29^ and anti-inflammatory effects^30^.

Before OPs and phenolic acid metabolites can affect gene expression in the host, they must be absorbed and distributed to target tissues and cells. In contrast, here we demonstrate that administration of non-absorbable PPs prevents HFHS-induced weight gain and reduces visceral adiposity, as well as modulating blood biological parameters and the expression of genes related lipid metabolism. Metabolomics in PP-treated mice identified several exogenous flavan-3-ol conjugates, which were also observed in the urine of OP-treated mice. However, our acute PP administration study suggested that these compounds were not degraded to flavan-3-ols, suggesting that most of the administrated PPs were not absorbed and were excreted via the cecum (data not shown). To investigate potential mechanisms, we also assessed a wide range of endogenous metabolites in PP-treated mice. Several markers of insulin resistance involving *p*-cresol and indole derivatives were identified in the urine of PP-treated mice, suggesting direct conversion from aromatic amino acids, tryptophan and tyrosine, by the gut microbiota^31^. *p*-Cresol production by microbes such as *Clostridium* in the large intestine is correlated with dietary protein and vegetable intake^32^. Rago *et al*. reported that lower levels of 3-indoxyl sulfate, *p*-cresol conjugates were found in the plasma following apple ingestion^33^. High levels of *p*-cresol and indoxyl acid are associated with an increased risk of cardiovascular disease, inflammation, and oxidative stress. In addition, bile acids influence absorption of dietary fats, lipid-soluble vitamins, and cholesterol in the intestine. Here, we showed that non-absorbed PPs prevent obesity and impaired lipid metabolism; these effects were associated with changes in the gut microbiota including the *Firmicutes*/*Bacteroidetes* ratio and modulation of endogenous metabolites. PPs with high molecular weight reach the colon almost unchanged and directly affect the gut microbiota in the intestine; therefore, further studies are required to determine the role of endogenous metabolites of PPs in the health benefits.

**Figure 1:**
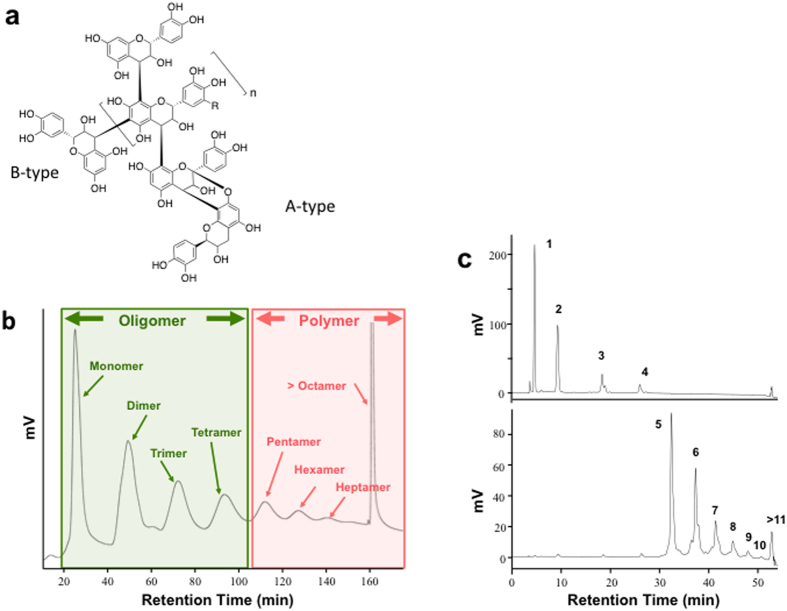
Chemical structure of procyanidins (**a**) and preparation of apple oligomeric procyanidins (OPs) and polymeric procyanidins (PPs) using normal-phase chromatography. Apple procyanidins were divided into two fractions using normal-phase chromatography (**b**) as described in the Materials and Methods section. The profiles of OPs and PPs were recorded (**c**). The chromatography conditions were as described in the Supplementary Methods. 1, monomer; 2, dimer; 3, trimer; 4, tetramer; 5, pentamer; 6, hexamer; 7, heptamer; 8, octamer; 9, nonamer; 10, decamer; 11, undecamer and larger.

**Figure 2:**
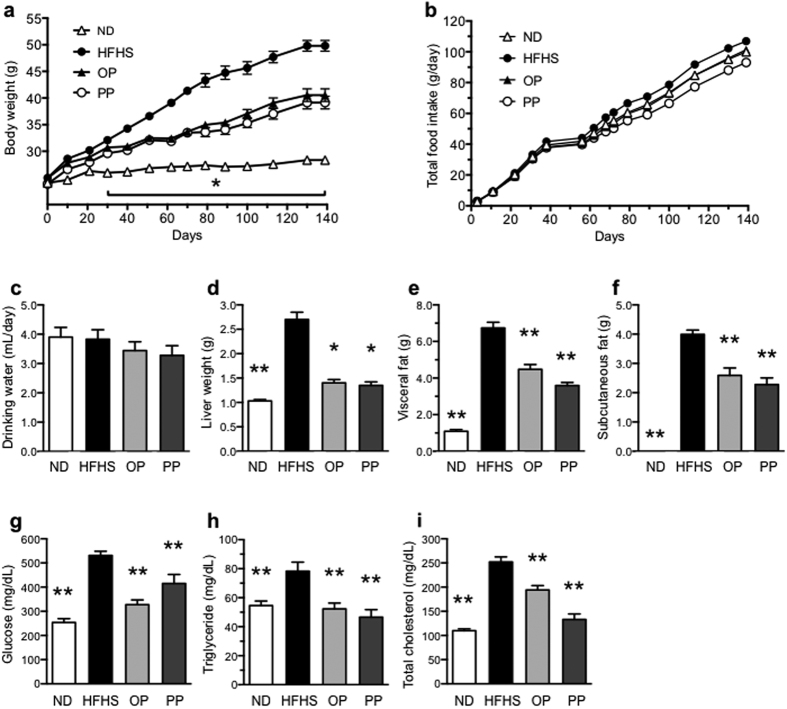
PP administration ameliorates negative changes in body composition. Body (**a**) and tissue ((**d**), liver; (**e**), visceral; (**f**), subcutaneous fat mass) weights were reduced in the HFHS-fed mice treated with PP for 20 weeks (*n* = 8), but no differences in food intake (**b**) or water consumption (**c**) were found. Data are expressed as the mean ± SEM (*n* = 8). Additionally, PP treatment prevented diet-induced increase in glucose (**g**), triglyceride (**h**), and total cholesterol (**i**) levels in the HFHS-fed mice after 20 weeks. *p < 0.05, **p < 0.01 versus the HFHS control group.

**Figure 3:**
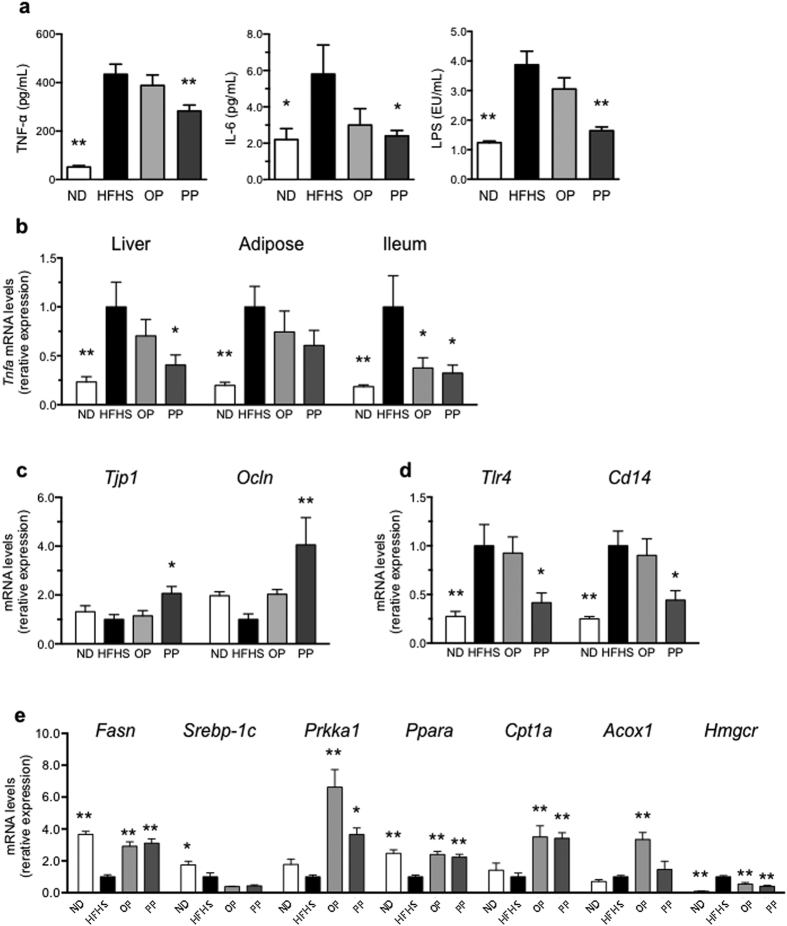
PP administration improves cytokine profiles, inflammatory markers and energy homeostasis. PP treatment prevented diet-induced increase in serum levels of TNF-α, and IL-6 (**a**) in the HFHS-fed mice after 20 weeks. Serum LPS (**a**) level and ileum permeability *Tjp1* and *Ocln* (**c**) in the mice treated with PP in comparison with those of HFHS-fed mice. PP treatment reduced gene expression of hepatic LPS receptor (*Tlr4* and *Cd14*) (**d**) and inflammatory cytokine *Tnfa* (**b**) in the HFHS-fed mice. Moreover, PP altered hepatic gene expression of lipid metabolism-related genes *Fasn* and *Hmgcr* as well as lipogenesis-related genes *Ppara*, *Prkaa1*, and *Cpt1a* in liver tissue (**e**). Values are expressed as the mean ± SEM (*n* = 8). *p < 0.05, **p < 0.01 versus HFHS control group.

**Figure 4:**
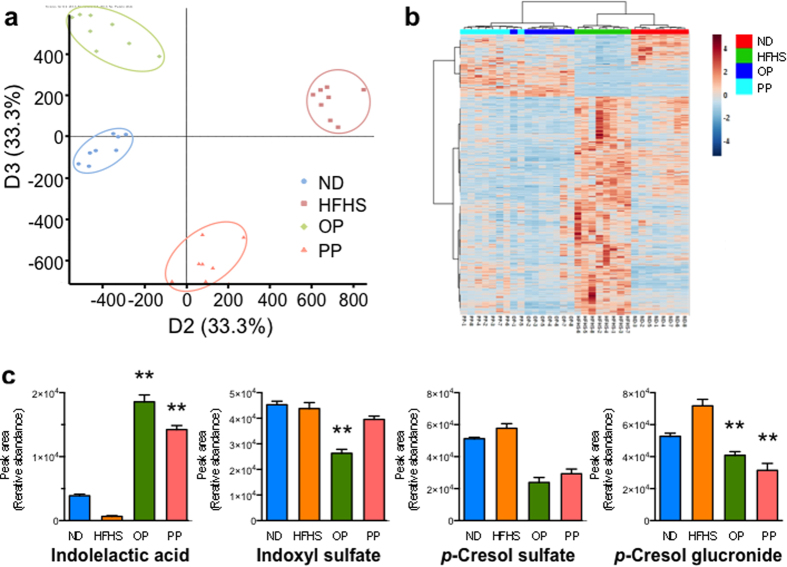
PP-treated mice urine metabolomes are distinct from those of HFHS diet-induced obesity. The score plot (**a**) and loading plot (Supplementary Fig. 1) obtained via principal component analysis-discriminant analysis of the urine metabolomes of mice treated for 20 weeks are shown. (**b**) Hierarchical clustering heat map of the urine metabolomes of each treatment group (*n* = 7–8), see Supplementary Table 1. (**c**) *p*-Cresol and indole derivatives in urine of C57BL/6J mice treated for 20 weeks. Values are presented as the mean ± SEM (*n* = 7–8). *p < 0.05, **p < 0.01 versus HFHS control group.

**Figure 5:**
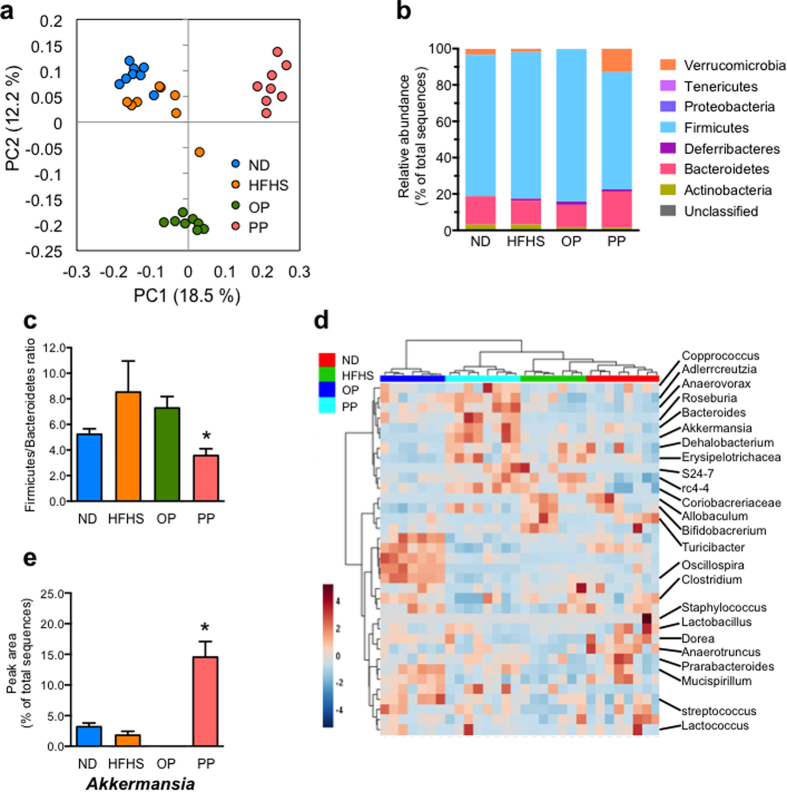
PP alters cecum microbial profiles in mice with HFHS diet-induced obesity. UniFrac-based principal co-ordinate analysis (**a**) showed that PP administration had a substantial effect on the gut microbial composition of HFHS-fed mice. The microbial composition (**b**) and *Firmicutes*/*Bacteroidetes* ratio (**c**) at the phylum level, hierarchical clustering heat maps (**d**), and microbial compositions (**e**) at the genus level of *Akkermansia* (calculated as percentage of total microbiota) of C57BL/6J mice treated for 20 weeks are shown. Values are presented as the mean ± SEM (*n* = 7–8). *p < 0.05, **p < 0.01 versus HFHS control group, see Supplementary Tables 2 and 3.

We demonstrated that PP-treatment attenuated the level of LPS and reduced intestinal permeability in the HFHS-fed mice. Administration of *Akkermansia* as a probiotic protected against obesity and prevented the HFHS diet-induced increase in LPS relaease^34^. Previous studies showed that administration of grape/red wine polyphenols^22^ or cranberry extract^21^ including procyanidins was associated with an increase in the proportion of *Akkermansia* in the gut microbiota of mice fed a high-fat diet. Anhe *et al*. reported that administration of cranberry extract improved metabolic syndrome and increased the relative proportion of *Akkermansia*, reduced intestinal permeability, and decreased circulating LPS levels^21^. Feliciano *et al*. reported that A-type procyanidins from cranberry were more effective in promoting extra-intestinal phathogenic *Escherichia coli* agglutination in comparison with B-type procyanidins from apple^35^. Cranberry procyanidins may decrease LPS level by the agglutination of the intestinal gram-negative bacteria including *Escherichia coli*. Our results also demonstrated that PP-administration reduced the levels of LPS and inflammatory IL-6 and TNF-α in the PP-treated HFHS-fed mice, as compared with the untreated HFHS-fed mice. Further studies indicated a reduction in the expression of genes related to the intestinal gut barrier (*Tjp1* and *Ocln*) and hepatic inflammation receptor (*Tlr4* and *Cd14*) in the PP-treated HFHS-fed mice. However, these studies did not elucidate which type of procyanidins is responsible for the various metabolic outcomes. Apple procyanidins are very similar compounds to cranberry and grape. Cranberry extract contains A-type procyanidin, which vary structurally with the formation of a second interflavanoid linkage by C-O oxidation coupling (Fig. 1a). Grape contains procyanidin B-type as well as prodelphinidins, which is one of proanthocyanidin according to its flavan-3-ol units, (+)-gallocatechin and (−)-epigallocatechin^36^. Importantly, our results are the first to suggest that non-absorbable PPs containing pentamers and larger polymers increased the proportion of *Akkermansia* in the gut microbiota of high-fat fed mice and that the degree of polymerization is important factor. Our results suggest that PP-mediated prevention of obesity and inhibition of lipid accumulation is associated with its prevention of gut barrier disruption or increased intestinal permeability to LPS. Further studies will analyze the increased proportion of *Akkermansia* in the PP-treated HFHS-fed mice.

By combining physiological evaluation and metagenomic/metabolomic profiling, the current study revealed that non-absorbable PPs alter lipid homeostasis in mice with diet-induced obesity. Red wine and oolong tea contain high-molecular weight polyphenols produced during the manufacturing process. Procyanidins in grapes and red wine are thought to reduce the risk of coronary heart disease and to lower overall mortality^37^. Corder *et al*. reported that procyanidins are present at higher concentrations in red wines produced by traditional production methods in regions of France where the population show a reduced risk of coronary heart disease^38^. Highly polymeric polyphenols in red wine may thus produce beneficial effects by altering the intestinal microbiota. Further studies will focus on the role of the gut microbiota in mediating the effects of highly polymeric polyphenols from plant. This may lead to the application of these non-absorbed compounds to the treatment of metabolic disorders.

## Methods

### Animals

Nine-week-old C57BL/6J male mice (*n* = 40; Charles River Japan Inc., Kanagawa, Japan) were housed in a controlled environment (5 mice per cage; temperature 25 ± 1 °C, humidity 60 ± 5%; 12/12-h light-dark cycle with lights on at 07:00) with free access to food and drinking water. After a week of acclimation with a normal diet (D12450K containing 10% kcal as fat; Research Diets Inc., New Brunswick, NJ), the mice were randomly divided into four groups; ND, HFHS, OP-treated HFHS, and PP-treated HFHS groups. Body weight gain, food intake, and drinking water amount were recorded three times per week. Urine was collected on the day before dissection. After 20 weeks, the mice were fasted for 3 h and sacrificed under pentobarbital. Blood was drawn and immediately centrifuged (3,000 rpm, 10 min). Adipose tissues (mesenteric, perirenal, testis, and subcutaneous fat pads), the liver, and the cecum were carefully dissected and immediately immersed in liquid N_2_ or RNA later^®^ (Ambion, Austin, TX, USA); they were then stored at −80 °C. The protocols approved by the Animal Ethics Committee of Kitasato University (Permit Number 13–120) and all experimental procedures were performed according to the guidelines of The National Agriculture and Food Research.

### Apple procyanidin

OP and PP fractions were prepared as described below. Briefly, the apple polyphenol fraction was separated from apple (*Malus pumila* cv. Fuji) juice using a preparative column with aromatic synthetic adsorbents and a Sepabeads^®^ SP-850 (Mitsubishi Kasei Co., Ltd., Japan) column (*i.d*. 80 × 1000 mm)^39^. APC fraction was purified in order to remove other flavonoids and polyphenols using a Diaion^®^ HP-20ss (Mitsubishi Kasei Co., Ltd., Japan) column (*i.d*. 57 × 1000 mm). After evaporation and lyophilization of the APC fraction, APCs were fractionated according to the degree of polymerization by preparative normal-phase chromatography using an Inertsil Prep-Silica (*i.d*. 50 × 250 mm) column (GL Science, Japan)^39^. A 2-g sample of the APCs fraction was dissolved in methanol (3 mL) and applied to the column. The flow rate was 50 mL/min. The mobile phase was a binary gradient composed of solvent A (hexane:methanol:ethyl acetate = 8:3:1, v/v/v) and solvent B (hexane:methanol:ethyl acetate = 3:3:1, v/v/v). For the first 30 min, the mobile phase was 100% solvent A, followed by a linear gradient from 0 to 95% solvent B for 150 min. The eluate was monitored by measuring the absorbance at 280 nm. APCs were separated into an OP fraction, which included monomers, dimers, trimers, tetramer, and a PP fraction that included pentamers and larger polymers (Fig. 1b). The OP and PP fractions were concentrated by rotary evaporation at 45 °C and lyophilized. The procyanidin profiles of these fractions are shown in Table 1. The procyanidin analysis method is described in the online Supplementary Methods. The chromatograms of the OP and PP fractions are shown in Fig. 1c.

### Blood biological parameters

Blood triglyceride, glucose, and total cholesterol levels were measured using a Fuji Dri-Chem 7000 instrument (Fuji Film, Tokyo, Japan). LPS levels were analyzed using a LPS kit based on a Limulus amebocyte extract (LAL kit QCL-1000^®^, Lonza, Switzerland). Serum TNF-α concentrations were determined using commercial enzyme-linked immunosorbent assay kit (Ready-Set-Go!^®^, eBioscience, San Diego, CA). Concentrations of IL-6 were determined in 12 μL of serum using a multiplex immunoassay kit (Bio-Plex Cytokine Assay, Bio-Rad Laboratories, Inc., CA, USA) and Bio-Plex^®^ Suspension Array System integrated with Bio-Plex Manager Software (version 6.0) (Bio-Rad). The high sensitivity setting was used for the reporter target channel (RP1) and fluorescent identification of the microspheres. Reporter conjugate emission wavelengths were maintained using a Bio-Plex^®^ Calibration Kit (Bio-Rad). The consistence of optical alignment, fluidics performance, doublet discrimination and identification of individual bead signatures was assessed using a Bio-Plex^®^ Validation Kit (version 4.0) (Bio-Rad). The coefficients of variation for bead discrimination and reporter channel identification did not exceed 7.0 and 8.0%, respectively.

### Quantitative real-time PCR

Individual tissue samples (20–30 mg) were homogenized for 20 sec with Precellys^®^ 24 (Bertin Technologies, Montigny le Bretonneux, France). Total RNA was prepared using the RNeasy Mini Kit (QIAGEN, Valencia, CA). cDNA was prepared by reverse transcription of total RNA using the Transcriptor Universal cDNA Master (Roche Diagnostics, Basel, Switzerland). Real-time quantitative PCR was performed with a StepOnePlus real-time PCR system (Applied Biosystems, Foster City, CA) using Fast SYBR^®^ Green Master Mix (Applied Biosystems) according to the manufacturer’s instructions. All samples were run in duplicate in a single 96-well reaction plate. Data were analyzed by the relative standard curve method. The identity and purity of the amplified products were checked by melting curve analysis. Primer sequences for the targeted mouse genes are presented in Supplementary Table 4. The relative amount of each target mRNA was normalized to the *Gapdh* mRNA level as an endogenous control gene in the same cDNA sample.

### Urine metabolomics using HPLC-QTOF/MS

Metabolomics measurements and data processing were performed as described in the online Supplementary Methods using a hybrid Q-TOF TripleTOF^®^ 5600 system (AB SCIEX, Concord, Ontario, Canada). First, 10 μL mouse urine was diluted with 40 μL of 50% acetonitrile and filtered (0.45 μm). Metabolite measurement was performed three times in negative mode in a randomized order to avoid possible bias. The chromatographic conditions for HPLC-QTOF/MS are described in the online Supplementary Methods. Alignment of the detected peaks was performed according to *m/z* value and normalized retention times using MarkerView^®^ Software (version 1.2.1, AB SCIEX). PCA, t-tests, and PCA-DA results were processed by MarkerView^®^ Software. Assignment of the spectral peaks (Supplementary Table 3) was performed using an in-house library, the Human Metabolome Database (HMDB; http://www.hmdb.ca/), and the Phenol-Explorer (http://www.phenol-explorer.eu/).

### 16S rRNA gene sequencing

After DNA extraction from the cecum^40^, the 16S rDNA V3-V4 region was amplified using TaKaRa Ex Taq HS DNA polymerase (TaKaRa Bio, Shiga, Japan) and 16S Amplicon PCR Primers (341F: 5′-*TCGTCGGCAGCGTCAGATGTGTATAAGAGACAG*CCTACGGGNGGCWGCAG-3′ and 785R: 5′-*GTCTCGTGGGCTCGGAGATGTGTATAAGAGACAG*GACTACHVGGGTATCTAATCC-3′). The italicized sequences in the primers are the Illumina overhang adapter^41^. PCR amplification was performed with the following program: an initial denaturation at 93 °C for 1 min; 20 cycles of 95 °C for 30 s, 55 °C for 30 s, and 72 °C for 30 s; and an additional extension at 72 °C for 5 min. The amplicons were adapted with Illumina sequencing adapters and dual-index barcode sequences using the Nextera DNA Library Preparation Kit (Illumina, San Diego, CA, USA), after which they were purified using the AMPure XP Beads Kit (Beckman Coulter, Inc., Brea, CA, USA) according to the manufacturer’s instructions. The purified amplicon library was sequenced on an Illumina Miseq platform (Illumina). Post processing sequencing data were analyzed using the Quantitative Insights into Microbial Ecology (QIIME) pipeline (version 1.8.0.)^42^. Quality filtering was performed using the default parameters in QIIME. Sequences were grouped into OTUs at a 97% sequence similarity threshold using UCLUST. Taxonomy was assigned using GreenGenes database^43^. The UniFrac pipeline in QIIME was used to evaluate beta diversity. PCoA was performed to plot the variation in the unweighted UniFrac distance between samples.

### Statistical analysis

All data are presented as the mean ± SEM. Statistical analysis was performed using one-way analysis of variance (ANOVA) followed by Bonferroni’s multiple comparison test, as well as using the Kruskal-Wllis non-parametric test with Dunn’s multiple comparison post-test. Body weight gain curve were statistically compared using a two-way repeated measures ANOVA followed by Bonferroni’s multiple comparison test (Graph Pad Prism^®^ version 6 for Macintosh). All results were considered statistically significant at *p* < 0.05.

### Ethics approval

Kitasato University Ethics Committee.

## Additional Information

**How to cite this article**: Masumoto, S. *et al*. Non-absorbable apple procyanidins prevent obesity associated with gut microbial and metabolomic changes. *Sci. Rep*. **6**, 31208; doi: 10.1038/srep31208 (2016).

## Supplementary Material

Supplementary Information

Supplementary Table 1

Supplementary Table 2

Supplementary Table 3

Supplementary Table 4

## Figures and Tables

**Table 1:** Characterization of apple procyanidin fractions.

	Oligomeric procyanidins (%)	Polymeric procyanidins (%)
Flavan-3-ol (monomer)	22.5	0
Procyanidin dimer	30.4	1.5
trimer	38.7	3.3
tetramer	8.4	2.1
pentamer	0	27.1
hexamer	0	23.2
heptamer	0	17.5
octamer	0	8.8
nonamer	0	3.1
undecamer	0	0.2
> dodecamer	0	6.2
