# Supplementary material for: Non-absorbable apple procyanidins prevent obesity associated with gut microbial and metabolomic changes
Source: Sci Rep. 2016 Aug 10;6:31208. doi: 10.1038/srep31208 (PMC4979010; doi:10.1038/srep31208)
Supplement: Supplementary Information [file srep31208-s1.pdf]

SUPPORTING INFORMATION

Title: Non-absorbable apple procyanidins prevent obesity associated with gut microbial and metabolomic changes

Authors: Saeko Masumoto,[1] Akari Terao,[2] Yuji Yamamoto,[2] Takao Mukai,[2] Tomisato Miura,[3] Toshihiko Shoji[1]

Afflications:

[1]National Institute of Fruit Tree Science, National Agriculture & Food Research, Tsukuba, Ibaraki, Japan

[2]Department of Animal Science, School of Veterinary Medicine, Kitasato University, Towada, Aomori, Japan

[3]Hirosaki University Graduate School of Health Sciences, Hirosaki, Aomori, Japan

Corresponding author:

Toshihiko Shoji, PhD

NARO Institute of Fruit Tree Science, The National Agriculture & Food Research, 2-1 Fujimoto, Tsukuba, Ibaraki 305-8605, Japan. Tel: +81-298-38-6470; Fax: +81-298-38-6437; e-mail: tshoji@affrc.go.jp

SUPPLEMENTARY METHODS

Procyanidin analysis

Apple procyanidins were filtered through a 0.45-µm PTFE syringe filter prior to injection into a Prominence HPLC system (Shimazu Corporation, Kyoto, Japan) equipped with a RF-20AXS fluorescence detector (Shimazu) and an Inertsil WP300 Diol (GL Science Inc., Tokyo, Japan) column (*i.d.* 4.6 × 250 mm; 5 µm). Mixtures of acetonitrile and acetic acid (mobile phase A, CH_3CN:HOAc = 98:2) and methanol, H_2O, and acetic acid (mobile phase B, MeOH:H_2O:HOAc = 95:3:2) were used as mobile phases. Elution was performed using a linear gradient of 0–7% B for 0–3.0 min, followed by a linear gradient of 7–30% B for 57.0 min. Subsequently, mobile phase B content was increased from 30% to 100% over 60.0–70.0 min. The mobile phase was subsequently returned to the initial conditions (0% B) to re-equilibrate for 10.0 min. The sample injection volume was 5 µL, the flow rate was set at 1.0 mL/min, and fluorescence detection of procyanidins was performed with excitation and emission wavelengths of 230 and 321 nm, respectively.

Metabolomic analysis by HPLC-QTOF/MS

Urine samples were analyzed by high performance liquid chromatography–quadrupole time-of-fright/mass spectrometry (HPLC-QTOF/MS) coupled with a hybrid Q-TOF TripleTOF® 5600 system (AB SCIEX) with a DuoSpray electrospray ionization (ESI) ion source and a Dionex UltiMate 3000 HPLC system (Dionex Corporation, Idstein, Germany). The column was an ACQUITY UPLC® BEH C18 (Waters Co., Milford, MA, USA) column (*i.d.* 2.1 × 50 mm; 1.7 µm) at 40 °C. A mixture of 0.1% formic acid in distilled water (mobile phase A) and 0.1% formic acid in acetonitrile (mobile phase B) was used as the mobile phase. The initial eluent was 0% B for 0–2.0 min, followed by a linear gradient from 0 to 50% B for 8.0 min. Subsequently,

mobile phase B was increased from 50% to 95% over 10.0–11.5 min. The mobile phase was returned to the initial conditions (0% B) to re-equilibrate for 3.5 min. The injection volume was 0.5 μL of the sample solution. The flow rate was set at 0.3 mL/min.

Data were acquired using an ion spray voltage of -4500 mV. The declustering potential was -80 V. The temperature was 650 °C. N_2 was used as the curtain (25 arbitrary units) and nebulizer (50 arbitrary units) gases. The instrument was set to perform one TOF/MS survey scan (150 ms) and 10 MS/MS scans (30 ms). MS acquisition was performed in negative ionization mode with a mass-to-charge ratio (*m/z*) of 120–1400 from 0 to 12 min. Mass accuracy was maintained by the use of an automated calibrant delivery system (AB SCIEX) interfaced to the second inlet of the DuoSpray source. The TOF was calibrated with Irganox 1010 (*m/z* 1175.7768; Sigma-Aldrich, St. Louis, MO, USA). The MS/MS analyses were performed with collision energy (CE) of -30 ± 15 V.

Data processing

The MS data were analyzed using MarkerView® Software (version 1.2.1, AB SCIEX). Peaks were identified for each sample, whereas alignment was performed using *m/z* values and retention times for the peaks for multivariate analysis. Peak finding was performed using a minimum spectral peak width of 1 ppm, a noise threshold of 5, and a subtraction multiple factor of 1.2. Alignment and filtering were used to ensure mass tolerance of 0.04 Da and retention time tolerance of 0.25 min.

Multivariate analysis

Several statistical analyses, including principal component analysis (PCA), t-tests, and PCA-discriminant analysis (PCA-DA) were performed using MarkerView® Software. The data were

visualized by constructing principal component scores and loading plots. Each point on the scores plot represented an individual urine sample, whereas each point on the loadings plot represented a single mass spectrometry data point.

Metabolite identification

Markers contributing to the discrimination of the different groups of rats were identified on the basis of exact mass, which was compared to the theoretical mass of expected metabolites. The data for each urine sample was subjected to isotope pattern-matched peak mining using the Extracted Ion Chromatogram Manager add-on for PeakView® Software (version 1.1.1, AB SCIEX). Assignment of the spectral peaks was confirmed using appropriate standards and mass fragmentation (MS/MS analyses). The Human Metabolome Database (HMDB; www.hmdb.ca) and Phenol-Explorer (http://www.phenol-explorer.eu/) database were queried by molecular formula to identify chemical structures.

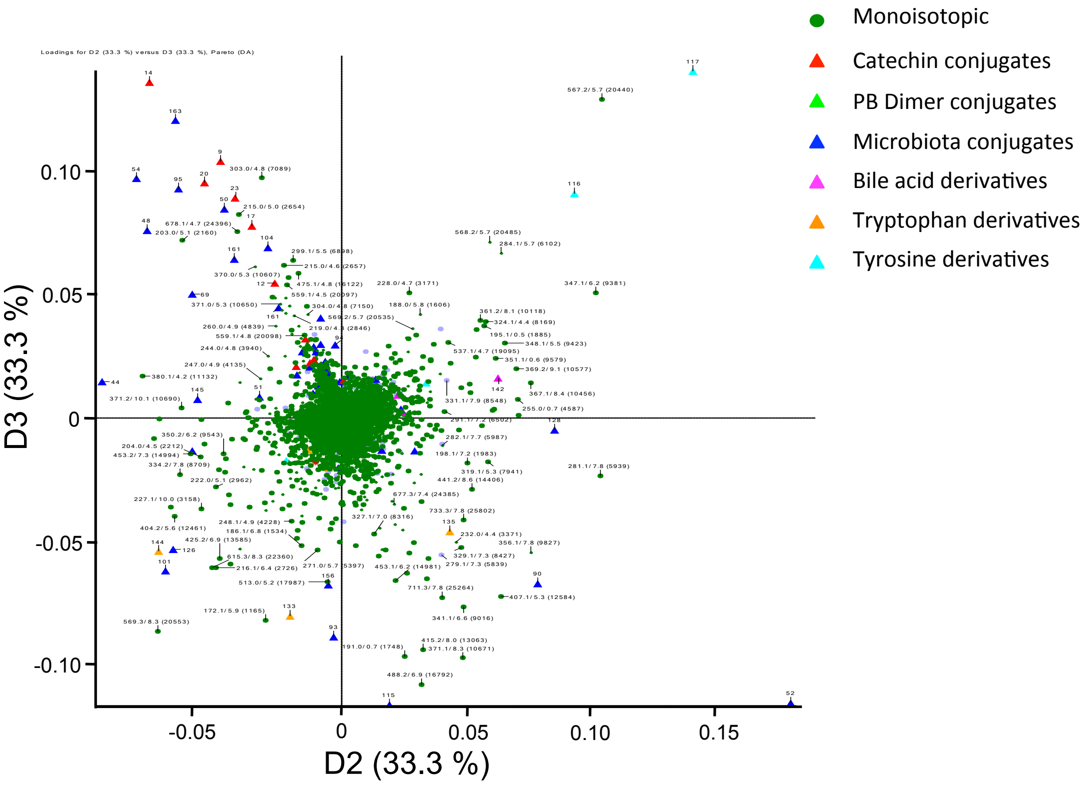

Supplementary figure 1. PP-treated mice urine metabolomes are distinct from those of HFHS diet-induced obesity mice. The loading plot obtained via principal component analysis-discriminant analysis (PCA-DA) of the urine metabolites of mice treated for 20 weeks are shown. Metabolomics by HPLC-QTOF/MS was performed by the method described in the online supplementary method.
